# Poor Prognosis of Advanced Gastric Cancer with Metastatic Suprapancreatic Lymph Nodes

**DOI:** 10.1245/s10434-012-2839-8

**Published:** 2013-01-09

**Authors:** Toru Kusano, Norio Shiraishi, Hidefumi Shiroshita, Tsuyoshi Etoh, Masafumi Inomata, Seigo Kitano

**Affiliations:** Department of Gastroenterological Surgery, Oita University Faculty of Medicine, Yufu, Oita, Japan

## Abstract

**Background:**

Whether gastrectomy with D2 lymphadenectomy improves survival of patients with advanced gastric cancer (AGC) remains controversial. Few studies have described the pathological features of AGC with metastatic suprapancreatic lymph nodes (LN), which are the target of D2 lymphadenectomy. This study therefore aims to clarify the prognosis and clinical pathological features including the number and location of metastatic LN in AGC with metastatic suprapancreatic LN.

**Methods:**

406 patients with AGC, who underwent gastrectomy with D2 lymphadenectomy from 1982 to 2007 at Oita University, were reviewed retrospectively with regard to presence or absence of metastatic suprapancreatic LN. The pathological factors associated with AGC with metastatic suprapancreatic LN were examined by univariate and multivariate analysis.

**Results:**

Of 362 patients with AGC, 78 had suprapancreatic LN metastasis (21.5 %), differing significantly in terms of presence of vascular invasion and having a larger number of metastatic perigastric LN in comparison with only metastatic perigastric LN on univariate analysis. According to multivariate analysis, they were associated with presence of vascular invasion and a large number of total metastatic LN (more than two; N2≤). The overall 5-year survival rate of the AGC with perigastric LN metastasis (station 1–7) group was 37.9 % and of the AGC with suprapancreatic LN metastasis group was 12.8 %. There were significant differences in each group (*P* < 0.05).

**Conclusions:**

Patients with AGC with metastatic suprapancreatic LN had a large number of total metastatic LN and poor prognosis, suggesting that it may be a systemic disease.

Gastric cancer is one of the most prevalent malignant tumors worldwide. Improvements in diagnostic modalities, such as endoscopy and barium examinations, have increased the incidence of early gastric cancer (EGC), whereas advanced gastric cancer (AGC) with poor prognosis has not decreased.

It is widely accepted that the most effective strategy for AGC is surgical treatment. The purpose of surgery for AGC is complete clearance of local cancer cells (R0), which involves both gastrectomy and lymphadenectomy. However, the optimal extent of lymphadenectomy for AGC is still under discussion. In Asian countries, gastrectomy with extended (D2) lymphadenectomy has been performed as a standard procedure for AGC, whereas gastrectomy with perigastric (D1) lymphadenectomy has been used in Western countries. Although randomized controlled trials to clarify the benefits of D2 lymphadenectomy for survival have been carried out in Germany, the UK, and Taiwan, a consensus has not been reached.^1^^–^ 9 Dissection of suprapancreatic lymph nodes (LN) is the main difference between D1 and D2 lymphadenectomy. However, few studies have described the pathological features or prognosis of AGC with metastatic suprapancreatic LN.

It was recently acknowledged that the total number of metastatic LN is a more reliable prognostic indicator than positive anatomical lymphatic stations.^10^^–^ 15 Therefore, in the tumor–node–metastasis (TNM) classification, the total number of metastatic LN was adopted to classify the stages of gastric cancer. Most AGC with more than six metastatic LN (N3) appear to be systemic disease because of poor prognosis, and the prognosis of AGC patients with metastatic suprapancreatic LN is known to be very poor, even after curative (R0) surgery.^16^ There is a possibility that they have a larger number of total metastatic LN.

The aim of this study is to clarify the prognosis and clinical pathological features including the number and location of metastatic LN in AGC with metastatic suprapancreatic LN.

## Patients and Methods

From January 1982 to December 2007, 362 patients with AGC underwent initial gastrectomy at the Department of Surgery I, Oita University Faculty of Medicine. Among them, we studied 165 patients with AGC with metastatic LN who underwent gastrectomy with D2 lymphadenectomy and with complete follow-up for 5 years after gastrectomy. The type of gastrectomy was determined according to the Japanese Guidelines for Diagnosis and Treatment of Carcinoma of the Stomach. Although we have introduced laparoscopy-assisted distal gastrectomy for such patients since 1994, the operative criteria have not been changed in our institution.^17^

The age and sex of the patients, the location, size, gross type, histological type, depth of wall invasion, and extent of lymphatic and vascular invasion of the AGC, and the number of positive LN were obtained from surgery and pathology records. These pathological findings were analyzed according to the Japanese Classification of Gastric Carcinoma, and the cancer stage was assessed according to the Union for International Cancer Control (UICC) TNM Classification of Malignant Tumors, 7th edition.^18^ To avoid sample bias, more than 14 LN were dissected from en bloc specimens and their classification was determined by surgeons and pathologists shortly after surgery, based on the Japanese Classification of Gastric Carcinoma. The presence of LN metastases was decided by pathologists using hematoxylin–eosin-stained specimens of a maximum section of the surface of the LN. The pathology records were reported by pathologists. We categorized suprapancreatic LN as those along the common hepatic artery (station 8a), around the celiac artery (station 9), along the proximal splenic artery (station 11p), and around the proper hepatic artery (station 12a).

First, the incidence of metastatic suprapancreatic LN in patients with AGC was reviewed. Next, the pathological features of AGC with metastatic suprapancreatic LN were determined. We divided AGC with metastatic LN into two groups: those with (*n* = 78) and those without (*n* = 87) metastatic suprapancreatic LN.

Postoperatively, patients were examined at follow-up visits every 3 months for the first 2 years and every 6 months thereafter. At each follow-up control, carcinoembryonic antigen and carbohydrate antigen 19-9 level were determined. Thoracicoabdominal and pelvic computed tomographic scan or abdominal ultrasonography was performed alternately every 3–6 months. Gastroscopy was performed yearly.

We then compared their pathological features using the Chi-square test. The Wilcoxon rank test was performed for median age and tumor size comparison. Multivariate analysis was used for adjusting the odds ratio and corresponding 95 % confidence interval. Cumulative probability of overall survival (OS) was estimated by Kaplan–Meier survival methods, and differences between subgroups were assessed by the log-rank test. Duration of follow-up was calculated as the time from surgery to the event of death. The reason for studying only OS was incomplete follow-up in some patients and consequent limitation of sample size as described above. Variables for which the *P* value on univariate analysis was less than 0.05 were included in subsequent multivariate analysis. *P* < 0.05 was considered to be statistically significant for all analyses.

All statistical analysis was performed using SPSS 11.0 statistics software. This study was conducted according to the Ethical Guidelines for Clinical Studies of Oita University Faculty of Medicine.

## Results

In this study, the incidence of AGC with metastatic LN was 75.7 % (165/218) of all AGC, with a mean number of metastatic LN of 9.9 ± 9.8. The rate of AGC with metastatic LN in the suprapancreatic area was approximately 35.8 % (78/218) of all AGC with metastatic LN, with a mean number of metastatic LN of 14.8 ± 11.6, which was significantly higher than that in AGC without metastatic suprapancreatic LN. Among these, 50 cases (64.1 %) had two or more metastatic LN in the suprapancreatic area, with a mean number of total metastatic LN of 18.8 ± 12.2. The incidence of metastatic suprapancreatic LN increased according to the depth of cancer: 7.7 % in T2 cancers, 25.6 % in T3, and 66.7 % in T4. Their frequency was not affected by the location of the cancer. The incidence of skip metastasis to suprapancreatic LN was only 2.6 % in AGC with metastatic LN.

**Fig. 1:**
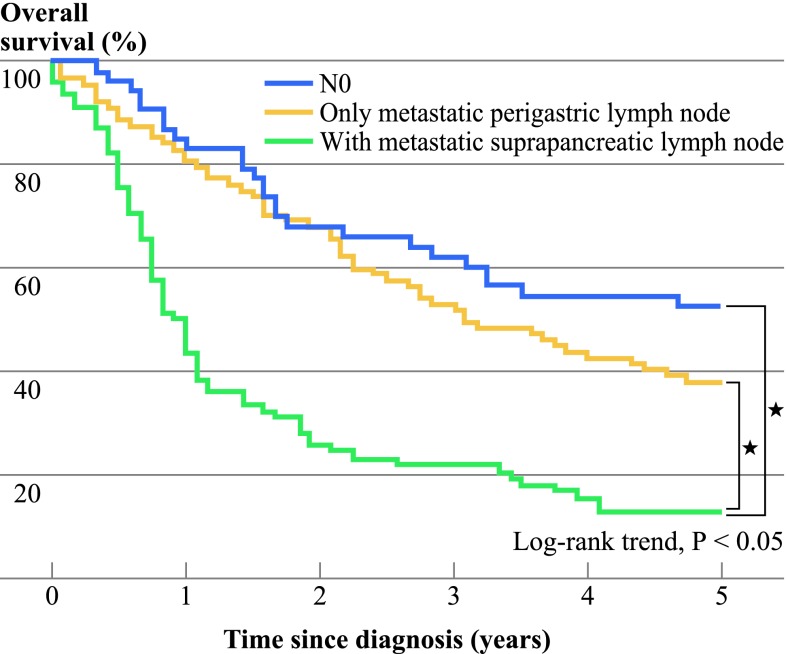
Kaplan–Meier survival analysis of advanced gastric cancer with no metastatic lymph nodes or with/without metastatic suprapancreatic lymph nodes (log-rank test). The 5-year OS was 52.8 % in patients with no metastatic lymph nodes (*n* = 53), 37.9 % in patients without metastatic suprapancreatic lymph nodes (*n* = 87), and 12.8 % in patients with metastatic suprapancreatic lymph nodes (*n* = 78). Survival rate after gastrectomy with D2 lymphadenectomy for AGC was significantly worse in patients with metastatic suprapancreatic lymph nodes than in those without (*P* < 0.05)

The median follow-up period of this study was 75 months. The median follow-up period was 74 months in the no metastatic LN (N0) group, 76 months in the perigastric LN metastasis (station 1–7) group, and 71.5 months in the suprapancreatic LN metastasis group. The overall 5-year survival rate of AGC in our study is shown in Fig. 1. The 5-year survival rate of the AGC without LN metastasis (N0) group was 52.8 % and of the AGC with only perigastric LN metastasis (station 1–7) group was 37.9 %; on the other hand, in the AGC with suprapancreatic LN metastasis group, it was 12.8 %. There were significant differences in each group (*P* < 0.05). Our study showed that the recurrence rates in the AGC with suprapancreatic LN metastasis group were 20.0 % for hematogenous metastasis, 5.0 % for metastasis to locoregional area, 25.0 % for paraaortic LN metastasis, and 50.0 % for peritoneal dissemination, whereas the recurrence rates in the AGC with only perigastric LN metastasis group were 24.3, 13.5, 13.5, and 48.7 %, respectively.

No significant differences were observed in patient sex or age or the surgical procedure between AGC with and without metastatic LN in the suprapancreatic area (Table 1), and the mean number of suprapancreatic LN harvested did not differ between the two groups. There was no significant difference in the location, size of tumor, gross type, histological type, depth of invasion, and extent of lymphatic invasion of the cancer between the groups. In the present study, the following pathological features of AGC with metastatic suprapancreatic LN were determined in comparison with AGC without such LN by univariate analysis: presence of vascular invasion, larger number of metastatic perigastric LN, and more advanced stage of cancer (Table 2). Detailed examination of AGC with metastatic suprapancreatic LN showed a larger number of total metastatic LN and also a larger number of metastatic LN along either the lesser curvature or the greater curvature (Table 3).

**Table 1:** Clinicopathologic features of patients with advanced gastric cancer with and without metastatic suprapancreatic lymph nodes

Clinicopathologic variable	Metastatic suprapancreatic lymph nodes		*P* value
	Present (*n* = 78)	Absent (*n* = 87)	
Sex			0.12
Male	59 (75.6 %)	56 (64.4 %)	
Female	19 (24.4 %)	31 (35.6 %)	
Age (years)			0.43
Median	65	66	
Range	32–83	28–84	
Operation method			0.70
TG	48 (61.5 %)	51 (58.6 %)	
DG	30 (38.5 %)	36 (41.4 %)	
Average dissected lymph nodes			0.95
Total number	35.5 (7–88)	32.3 (8–67)	
Lesser curvature (station 1, 3, 5, 7)	13.8 (2–37)	14.3 (0–30)	
Greater curvature (station 2, 4, 6)	11.7 (0–27)	11.1 (0–33)	
Suprapancreatic lymph nodes	10.0 (1–28)	6.9 (1–23)	

*TG* total gastrectomy, *DG* distal gastrectomy

**Table 2:** Pathological features of the tumor in advanced gastric cancer with and without metastatic suprapancreatic lymph nodes

Clinicopathologic variable	Metastatic suprapancreatic lymph nodes		*P* value
	Present (*n* = 78)	Absent (*n* = 87)	
Location			0.39
U	24 (30.8 %)	33 (38.0 %)	
M	32 (41.0 %)	27 (31.0 %)	
L	22 (28.2 %)	27 (31.0 %)	
Tumor size (mm)			0.091
Median	73.5	62	
Range	28–195	18–220	
Gross type			0.35
Type 1 + 2	25 (32.1 %)	34 (39.1 %)	
Type 3+4	53 (67.9 %)	53 (60.9 %)	
Histological type			0.28
Well/moderately	25 (32.1 %)	35 (40.2 %)	
Poorly	53 (67.9 %)	52 (59.8 %)	
Depth of invasion			0.090
T2	6 (7.7 %)	13 (14.9 %)	
T3	20 (25.6 %)	32 (36.8 %)	
T4	52 (66.7 %)	42 (48.3 %)	
Lymphatic invasion			0.39
Present	75 (96.2 %)	81 (93.1 %)	
Absent	3 (3.8 %)	6 (6.9 %)	
Vascular invasion			<0.05
Present	50 (64.1 %)	41 (47.1 %)	
Absent	28 (35.9 %)	46 (52.9 %)	
Lymph node metastasis			<0.01
N1	3 (3.8 %)	30 (34.5 %)	
N2	18 (23.1 %)	30 (34.5 %)	
N3	57 (73.1 %)	27 (31.0 %)	
N3a	28	22	
N3b	29	5	
UICC stage			<0.01
IIA	1 (1.3 %)	6 (6.9 %)	
IIB	3 (3.8 %)	18 (20.7 %)	
IIIA	11 (14.1 %)	19 (21.8 %)	
IIIB	8 (10.3 %)	19 (21.8 %)	
IIIC	55 (70.5 %)	25 (28.8 %)

**Table 3:** Number of perigastric lymph nodes in advanced gastric cancer with and without metastatic suprapancreatic lymph nodes

Clinicopathologic variable	Metastatic suprapancreatic lymph nodes		*P* value
	Present (*n* = 78)	Absent (*n* = 87)	
Total number of metastatic lymph nodes			0.13
Perigastric lymph nodes (station 1–7)			
Present	76 (97.4 %)	87 (100 %)	
Absent	2 (2.6 %)	0 (0 %)	
Number			<0.01
*n* = 0	2 (2.6 %)	0 (0 %)	
1 ≤ *n* ≤ 2	8 (10.3 %)	30 (34.5 %)	
3 ≤ *n* ≤ 6	17 (21.7 %)	30 (34.5 %)	
7 ≤ *n* ≤ 15	33 (42.3 %)	22 (25.3 %)	
16 ≤ *n*	18 (23.1 %)	5 (5.7 %)	
Lesser curvature (station 1, 3, 5, 7)			0.80
Present	70 (89.7 %)	77 (88.5 %)	
Absent	8 (10.3 %)	10 (11.5 %)	
Number			<0.01
*n* = 0	8 (10.3 %)	10 (11.5 %)	
1 ≤ *n* ≤ 2	14 (17.8 %)	31 (35.6 %)	
3 ≤ *n* ≤ 6	25 (32.1 %)	34 (39.1 %)	
7 ≤ *n* ≤ 15	24 (30.8 %)	10 (11.5 %)	
16 ≤ *n*	7 (9.0 %)	2 (2.3 %)	
Greater curvature (station 2, 4, 6)			<0.01
Present	65 (83.3 %)	49 (56.3 %)	
Absent	13 (16.7 %)	38 (43.7 %)	
Number			<0.01
*n* = 0	13 (16.7 %)	38 (43.7 %)	
1 ≤ *n* ≤ 2	22 (28.2 %)	28 (32.2 %)	
3 ≤ *n* ≤ 6	19 (24.4 %)	11 (12.6 %)	
7 ≤ *n* ≤ 15	23 (29.4 %)	10 (11.5 %)	
16 ≤ *n*	1 (1.3 %)	0 (0 %)	

Subsequent multivariate analysis using these significant factors showed that presence of vascular invasion and larger number of metastatic LN (more than two nodes; N2≤) were associated with AGC with metastatic suprapancreatic LN (Table 4).

**Table 4:** Results of multivariate analyses of clinicopathologic factors in advanced gastric cancer with and without metastatic suprapancreatic lymph nodes

	Odds ratio (95 % CI)	*P* value
Vascular invasion		<0.05
	2.201	
	(1.07–4.52)	
Total number of metastatic lymph nodes		<0.01
N2≤	9.841	
	(2.77–34.97)	

*CI* confidence interval

## Discussion

Whether gastrectomy with D2 lymphadenectomy improves survival of patients with AGC remains controversial. In Asian countries, gastrectomy with D2 lymphadenectomy has been performed as a standard procedure for AGC, whereas in Western countries, gastrectomy with D1 lymphadenectomy has been performed. Dissection of suprapancreatic LN is the main difference between D1 and D2 lymphadenectomy. Because only a few studies have examined them previously, we investigated the prognosis and clinical pathological features of AGC associated with metastatic suprapancreatic LN.

The present study showed that suprapancreatic LN metastasis occurred in approximately 35.8 % of AGC with metastatic LN, with a mean number of total metastatic LN of 14.8 ± 11.6. Subsequent multivariate analysis showed that presence of vascular invasion and LN metastasis (N2≤) were independent pathological factors associated with AGC with metastatic suprapancreatic LN. Thus, most AGC with metastatic suprapancreatic LN appear to be at a very advanced stage.

This study was retrospective and was conducted at a single institution. That is why we had to exclude 197 cases whose follow-up was not complete. In addition, analysis of the survival curve included only OS because of sample size limitation and incompleteness of recurrence data. In the present study, the quantity of metastatic cancer cells in each LN was not evaluated, and micrometastasis and extranodal metastasis were not examined.

Many studies have been conducted in Japan on the flow of lymphoid fluid and the distribution of the LN along the stomach to determine how gastric cancer cells spread through lymphatic vessels.^19^^–^ 21 Cancer cells from the primary lesion flow through the lymphoid vessels and produce an embolus in the LN, after which metastatic LN are formed according to a regular probability. Lee et al. showed that skip metastasis to the suprapancreatic LN occurred in 2.8 % of EGCs.^22^ In the present study, the incidence of skip metastasis to the suprapancreatic LN was only 2.6 % in AGC. Therefore, the frequency of skip metastasis in AGC and EGC was similar. Skip metastasis may be a phenomenon that occurs relatively early with a small number of cancer cells in the lymphatic vessel.

The risk factors for LN metastases in gastric cancer have been identified as the depth, size, and histological type of cancer.^23^^–^ 25 In the seventh edition of the UICC classification, the number of metastatic LN was adopted as an index of the stages of a disease. Katai et al. reviewed the characteristics of LN metastasis in 1,230 gastric cancers, including EGC, and showed that gastric cancer with metastatic suprapancreatic LN was associated with a large number of metastatic perigastric LN (>3 positive nodes).^26^ Our result that a larger number of metastatic LN (N2≤) was associated with AGC with metastatic LN supports their study. Adachi et al. showed that the prognosis of patients with two or more metastatic suprapancreatic LN, including those around the left gastric artery, was extremely poor.^16^ Our preliminary data show that a considerably higher number of total metastatic LN (N3b) is associated with AGC with two or more metastatic suprapancreatic LN (data not shown).

Our data showed extremely poor prognosis in the AGC with suprapancreatic LN metastasis group. The 5-year survival rate in this study was 12.8 % for the AGC with suprapancreatic LN metastasis group. According to the annual report of the Japanese Gastric Cancer Association (JGCA), 2008, 13th edition, the 5-year survival rates for AGC were 91.9 % for stage IA, 85.1 % for stage IB, 73.1 % for stage II, 51.0 % for stage IIIA, 33.4 % for stage IIIB, and 15.8 % for stage IV.^27^ According to the JGCA criteria, T4 ± N0, T3 + N1, and T2 + N2 constitute stage IIIA whereas T4 + N1 and T3 + N2 constitute stage IIIB. However, our study included these cases and stage IV cases with grading such as T2 + N3, T3 + N3, T4 + N2, and T4 + N3. We consider that the difference in criteria between JGCA and our study was the reason for the poor prognosis in this study.

Some studies have reported the recurrence patterns of gastric cancer. Ho et al. reported that the recurrence rates of EGC with metastasis to distant, locoregional, and peritoneal area were 55.7, 34, and 10.3 %, respectively.^28^ The liver was the most common site of recurrence in distant metastasis. Moriguchi et al. reported that the recurrence rates of AGC with metastasis to distant, locoregional, peritoneal, and other sites were 35.7, 11.3, 31.5, and 21.5 %, respectively.^29^ In our study, the locoregional recurrence rate in the perigastric LN metastasis group was similar to the rate observed in the study by Moriguchi et al. However, our data showed that there were very few locoregional recurrences in the suprapancreatic LN metastasis group. The peritoneal area was the most common site of recurrence, and the second most common site was the paraaortic LN area in the suprapancreatic LN metastasis group. These findings suggest that AGC with suprapancreatic LN metastasis has poor prognosis.

Whether gastrectomy with D2 lymphadenectomy improves survival of patients with AGC compared with gastrectomy with D1 lymphadenectomy is controversial. The Dutch trial and British Medical Research Council trial failed to show a benefit of gastrectomy with D2 lymphadenectomy for survival, but rather showed that this procedure increased the rate of complications, such as pancreatic juice.^1^^–^ 5,7 ^,^ 8 Macroscopic metastasis and micrometastasis to the LN around the left gastric artery (station 7) and suprapancreatic area might be causes of such a result. D1 lymphadenectomy in these studies is perigastric LN dissection, and it is different from D1 lymphadenectomy in the latest Japanese gastric cancer treatment guidelines (2010).^27^ In other words, D1 lymphadenectomy includes station 7 in the Japanese gastric cancer treatment guidelines of 2010. Station 7 is LN in the mesentery, and it is necessary to examine the significance of metastasis to station 7. On the other hand, a randomized trial in Taiwan demonstrated an improved survival of 6 % after D2/3 lymphadenectomy compared with D1 lymphadenectomy.^9^ Moreover, according to the long-term (15 years) results of a randomized nationwide Dutch D1/D2 trial, not OS but the disease-free survival rate was shown to improve after D2 lymphadenectomy.^30^ Therefore, the guidelines of the European Society for Medical Oncology and the National Comprehensive Cancer Network have been amended to recommend D2 lymphadenectomy as the standard treatment procedure. When metastasis to suprapancreatic LN in AGC is unclear by preoperative diagnosis and if the operative complications of D1 and D2 lymphadenectomy are similar, D2 lymphadenectomy is recommended as a standard procedure. However, for AGC with defined suprapancreatic LN metastasis, preoperative chemotherapy might be recommended.

In this study, metastatic suprapancreatic LN were examined and station 7 was not examined. The presence of more than two total metastatic LN and vascular invasion were associated with AGC with metastatic suprapancreatic LN, which accounted for 35.8 % of all AGC. AGC with metastatic suprapancreatic LN had a larger number of total metastatic LN, suggesting that the prognosis of patients with this disease was extremely poor. This may be a reason why it is difficult to prove the benefits of gastrectomy with D2 lymphadenectomy for survival of patients with AGC. Therefore, further multicenter studies are required to confirm the prognosis and clinicopathological characteristics of AGC with metastasis to suprapancreatic LN. Additionally, micrometastasis, the molecular biological characteristics of AGC with metastatic suprapancreatic LN, and circulating cancer cells are required to clarify the clinical significance of suprapancreatic metastasis in the near future.
